# Exploring NursEs lived Experience of Discussions about Sexual health, with kidney patients in Devon (NEEDS)

**DOI:** 10.1002/nop2.152

**Published:** 2018-06-01

**Authors:** Maxine Hough, Maggie Shepherd, Rohan Chauhan, Roy Powell, Jenny Childs

**Affiliations:** ^1^ Royal Devon and Exeter NHS Foundation Trust Exeter UK; ^2^ University of Exeter Medical School Exeter UK; ^3^ University of Saint Mark and Saint John Plymouth UK

**Keywords:** communication, conversations, nurse, qualitative approaches, renal nurse, sexual health

## Abstract

**Aim:**

The aim of the study is to gain a better understanding about the complexities of discussing sexuality with patients.

**Design:**

The study will explore how nurses understand the phenomenon by using an Interpretive Phenomenological Approach; the technique encourages deep reflection and rich descriptions about lived experience.

**Methods:**

Six nurses will be interviewed using iterative, open‐ended questions with prompts to illicit rich data.

**Predicted Results:**

Understand the support required to enable nurses to engage in deeper, more fulfilling conversations with patients about sexuality.

**Why this study is needed:**

- To gain a greater understanding about the complexities of discussing sexuality with patients.
- To establish what support is required to help renal nurses to deliver an effective management strategy for this problem.

## 1 BACKGROUND INFORMATION

It is estimated that 5% of the UK population have Chronic Kidney Disease (CKD) (Renal Organisation 2013). Sexual dysfunction (SD) in this group is well documented (Frazao, Bezerra, & Paiva, 2013; Strippoli, 2012; Toorians, Giltay, Donker, & Gooren, 1997) and most notably related to side effects of medication and renal replacement therapy, diabetes, vascular disease and hormonal dysregulation (Macready, 2008; van Ek et al., 2015). CKD patients have reported increased prevalence of erectile dysfunction, lack of libido, difficulty reaching orgasm, poor vaginal lubrication and loss of sexual desire and fertility (Navaneethan et al., 2010; Vecchio et al., 2010).

Studies suggest that care providers predominantly focus time and resources on renal replacement therapy and physical symptom management rather than mental health, body image and self‐esteem issues (Almutary, Bonner, & Douglas, 2013). Concentrating on life‐saving treatments when faced with this dichotomy is understandable in a health service with limited resources. However, it is not surprising then, that this patient group compared with the general population has significantly reduced quality of life scores (Vecchio, Palmer, Berardis, Craig, & Johnson, 2012), more diagnoses of anxiety disorder (Cohen, Cukor, & Kimmel, 2016 *)* and a higher prevalence of depression (Bohra & Novak, 2015).

There is a little research exploring this phenomenon; however, a study of male and female dialysis patients showed significantly reduced quality of life factors which appeared to actively correlate to increased levels of sexual dysfunction (Azevedo et al., 2014). Quinn, Happell, and Welch (2013) described nurses getting too narrowly focussed on illness and medications, having personal fears and feeling “uncomfortable” discussing sexuality and body image with patients. Saunamaki and Engstrom (2013) found that a large cohort of nurses feel that it is the physician's responsibility to manage a patient's sexuality or that the subject is “Taboo.”

In 1980, Roper Logan and Tierney presented the nursing profession with a gold standard tool to engage with patients and holistically assess needs and plan care (Roper, Logan, & Tierney, 1980). Sadly, “expressing sexuality” is an activity of daily living which has long been neglected by the profession.

With this in mind against a backdrop of an ever‐expanding CKD community, there has never been a better time for renal nurses to engage in therapeutic and meaningful dialogue about sexuality with their patient. It is clear from the lack of literature, especially in the UK; there is an obligation to study this phenomenon in greater detail and to collect rich and rigorous data from nurses involved in the care of these patients.

Current research in this area, although qualitative, remains superficial; it has yet to adequately define the “lived experience” of these phenomena. This study will examine what components underpin the barriers that are preventing nurses from adequately attaining the level of intimacy and communication required to have a professional and therapeutic conversation about sexuality. The results of this will help guide and inform professional practice, educators and policy makers.

## 2 PURPOSE

The purpose of this study is to gain a greater understanding about the complexities of discussing sexuality with patients and to establish what support is required to help renal nurses in Exeter to deliver an effective management strategy for the problem.

## 3 AIMS AND OBJECTIVES

- To describe how renal nurses feel about discussing sexuality with their patient.
- Explore the nurse's emotional response to the problem.
- To understand the essence of the “lived experience” of dealing with sexual conversations.

## 4 STUDY DESIGN

The study aims to explore how individuals experience a shared phenomenon, so a qualitative method has been chosen. Qualitative research is deemed the most effective methodology to understand and explain social phenomenon from the inside (Flick & Gibbs, 2007).

The study is underpinned by a constructivist paradigm; researchers would not use established theories but will seek to understand the complexity of participant's thoughts through discussion and interaction (Creswell, 2013). An Interpretive Phenomenological Approach (IPA) will be employed to encourage deep reflection in participants. The phenomenological aspect involves empirically using an individual's experience to obtain rich descriptions and understand the essence of the experience. The interpretive aspect will seek to decipher meaning and produce theories from the bottom–up. This is rooted in Husserl's “philosophical science of consciousness” (Husserl, 1982) and Hermeneutics (Byrne, 2001; Smith, Flowers, & Larkin, 2009). Husserl's theory argues that access to the material world is through consciousness and that all knowledge can be derived from experience. Hermeneutics theory of interpretation assumes that humans experience the world through language, which provides both understanding and knowledge. The study will adopt an epistemological viewpoint whereby knowledge is generated by gaining an intimate connection with the participant's inner world using deep conversational analysis and interpretation.

Interpretive Phenomenological Approach is particularly useful when discussing sex and sexuality, it uses an inductive approach to ask the expert (the participant) to talk about problems rather than the researcher making assumptions and relying on their own pre‐defined theories (Smith et al., 2009). Attaining a deeper knowledge about experiences in health care and interpreting meanings from how these are expressed can significantly influence health behaviour (Pringle, Drummond, McLafferty, & Hendry, 2011).

## 5 METHOD

Lightly structured interviews will be used with an iterative process informing the questions (Cohen et al., 2016). Interview questions may change and develop using themes brought up by early participants to be built on or carried forward to subsequent interviews. There will be two or three core questions (See Appendix A) and prompts will be used to encourage illustration of lived experiences and generate rich and honest conversation.

Questions will be piloted with two volunteer renal nurses before the study to assess feasibility and validity; this will ensure that any ambiguous and unreasonable language is discarded or revised beforehand. Interviews will be recorded on an audio recording device. Moreover, a second researcher will be used during the interview to take field notes, observe and ensure a confirmable record of proceedings.

## 6 STUDY SETTING

The qualitative, interview study will be conducted in one renal unit site in Devon, this will be:

- The Royal Devon and Exeter NHS Foundation Trust

## 7 RECRUITMENT STRATEGY

Promotional materials will be used, and ward managers will be approached to refer potential participants. First contact will be via a phone conversation or letter of invitation. A participant information sheet will be posted to potential participants. Respondents will be asked to confirm their interest by adding contact details and returning an affirmation slip in the stamped addressed envelope provided. Potential participants will be assessed for eligibility and contacted via telephone or face‐to‐face to discuss the study. If respondents confirm that they would like to take part, a mutually convenient time will be arranged to perform the interview.

## 8 SAMPLE

To ensure the most extensive range of participants possible, a purposive strategy will be used to target under‐represented groups. This will ensure that the cohort includes male and females of differing ages. Researchers anticipate interviewing a sample of six renal nurses, but this number would be informed by principles of data saturation (Creswell, 2013).

## 9 INCLUSION CRITERIA

- Registered nurses working regularly with kidney disease patients in Exeter.
- Nurses managing the care needs of kidney disease patients from admission to discharge and/or follow‐up at home.

## 10 EXCLUSION CRITERIA

- There are no exclusion criteria for this study.

## 11 INFORMED CONSENT

Participation is voluntary; the researcher will discuss the study with the respondent and answer any questions. The respondent will be advised that they are free to withdraw from the study at any time, but if they do so, the researcher will ask for their permission to use any data collected until that point. The respondent will be asked to sign a consent form. A copy of the signed consent form will be given to the respondent; the original will be kept in the study master file.

## 12 RIGOUR AND REFLEXIVITY

Reflexivity will be employed by the Chief Investigator to maximize rigour, this will increase the confidence and credibility of findings (Darawsheh, 2014):

- The researchers will “bracket” her pre‐existing theories so that the interviewee's insights will have sole focus (McCabe & Holmes, 2009; Smith et al., 2009).
- The researchers aim to control inherent experimenter bias by managing subconscious use of subtle clues in body language or tone of voice which may influence the subject into giving answers skewed towards the interviewer's own opinions and prejudices (Strickland & Suben, 2012). To control this, the interviewer will maintain a neutral demeanour throughout the interview.
- The chief investigator will keep a personal comprehensive and continuous reflexive diary. This will support the data and leave an ongoing “super audit trail” detailing moral and social stance (Rolfe, 2006). This will be published in partnership with findings for quality to be adequately appraised.

## 13 CREDIBILITY

To ensure the accuracy of findings, the use of prolonged engagement and persistent observation will be adopted (Altheide & Johnson, 1994). The researcher will ensure sufficient time in the field to gain full understanding of the phenomena under investigation. Interviewing will continue until there is no new emerging data evident and until saturation has been achieved (Houghton, Casey, Shaw, & Murphy, 2013).

## 14 TRANSFERABILITY

To ensure transferability, the original narrative will be sufficiently chronicled so that judgements can be made. In this way, readers will be able to make informed decisions about the transferability of the findings to their specific contexts (Lincoln & Guba, 1985). The study will concentrate on creating “thick” descriptions and giving examples of raw data so that interpretations can be made (Pringle et al., 2011). The researcher plans to present rich and robust results with direct quotations to substantiate findings. Additionally, excerpts from field notes will provide evidence of how themes were interpreted from the data (Houghton et al., 2013).

## 15 CONFIRMABILITY

Rigour and trustworthiness will be demonstrated by documenting honest descriptions discernible to readers. Evidence in the form of comprehensive reflexive and field notes related to the contextual background of the data will be maintained, alongside researcher rationale for all methodological decisions. Moreover, notes taken by a second researcher will be presented alongside transcribed audio data. These triangulated findings should optimize the validity of the results (Creswell, 2013).

## 16 DATA ANALYSIS

Analysing the data acquired during the study will be a fluid “back and forth” process based on Heidegger's Hermeneutic circle (Heidegger, 1962). (See Appendix B) To achieve this, the following strategies will be employed:

### 16.1 Reading and rereading

Data will be transcribed, and then, the researchers will become immersed in the narrative, entering the world of the participant by reading and rereading whilst imagining the voice of the individual. This will ensure that the participant is the sole focus of the inquiry (Smith et al., 2009).

### 16.2 Initial noting

The next stage will involve noting areas of interest after becoming intimately familiar with the text. The researchers will engage with the transcript and examine the participants true meaning, a comprehensive set of notes and comments will be compiled by exploring:

#### 16.2.1 Description

Here, the researchers will unpick key words and phrases which matter to the participant, descriptions of events and experiences which structure the participant's feelings towards discussing sexuality will be identified (Smith et al., 2009).

#### 16.2.2 Linguistics

There will be a focus on how language was presented, for example, was the participant laughing when they made the comment? Were there long pauses and what did that mean? Were metaphors used to describe experiences or feelings?

#### 16.2.3 Conceptualization

Professional experience, creativity, inductive reasoning and intuitive processes will be used to interpret data and understand it (Smith et al., 2009).

Following exploratory noting, the researchers will identify emerging phrases from research notes (not the transcripts) based on the objectives of the study. Phrases will be condensed and organized into categories and given a code. Ultimately, themes will be extrapolated using horizonalization, that is, a reflection of original words which disclose the full experience and the researcher's interpretation and understanding of these (van Manen, 1990). In line with the Consolidated Criteria for Reporting Qualitative Research (COREQ) recommendations, findings will be returned to participants for confirmation and feedback (Tong, Sainsbury, & Craig, 2007).

## 17 CONFIDENTIALITY AND DATA PROTECTION

Respondents will be known to the researcher; all recordings and documentation will be stored in an anonymized case report file (CRF) with a unique identifier. CRF's will include recruitee's initials and personal number. All participants will be assured of the confidentiality of the data collected, but will asked during the consent process for their permission to publish anonymized quotations from the study. Demographics collected will include age, gender and religion. CRF's will be stored in a locked cupboard, information collected will not be shared with other groups, the respondent will be informed that published results may be used by other researchers or outside organizations, but data are anonymized and not traceable to the individual.

## 18 STRENGTHS AND LIMITATIONS

Maxine Hough has significant clinical and research experience in renal nursing which will reinforce study rigour and trustworthiness. This expertise will strongly support the conceptual analysis of data. The obvious limitation of the study is the sensitive subject matter which may cause apprehension in potential participants. To avoid attrition researchers will use excellent interpersonal skills to ensure that participants are fully supported. Consideration will be given to avoid unnecessary time inconvenience and burden on the workplace. Moreover, researchers will present findings to all those involved in the recruitment phase as well as to the participants (Patel, Victor, & Lakshika, 2003).

## 19 ANTICIPATED RESULTS

The researchers do not hold any preconceived ideas about what the results of this research will offer, as this goes against the aims of this research approach, which is to fully understand this issue from the point of view of the nurses experiencing it. However, it is anticipated that resulting data will be used to inform further research to explore this phenomenon and in the development of interventions, educational tools and policy to support renal nurses engage in more fulfilling conversations with their patients about sexual health problems. In this way, renal care providers will truly be able to deliver first class, patient‐centred care, tailored to meet the needs of this client group.

## 20 STUDY DATES

Proposed Recruitment start date: October 2017.

Proposed Recruitment end date: April 2018.

## 21 PATIENT AND PUBLIC INVOLVEMENT

The study questionnaire has been designed in collaboration with members of the Royal Devon and Exeter PPIe group. Three current renal patients have informed the questions to be used during interviews with participants.

## 22 ETHICAL CONSIDERATIONS

The research team will complete a delegation log which will clearly define roles; this will be kept in the master file along with up‐to‐date GCP certificates and curriculum vitae for all study personnel. The master file and CRF's will be maintained by those delegated to do so and as per GCP guidelines (National Institute for Health Research Clinical Research Network (NIHR CRN), 2014). The master file will be stored in a locked research cupboard on site at the Royal Devon and Exeter Hospital.

Researchers do not have to apply to the Research Ethics Committee as no patients will be approached during this research.

## 23 DISSEMINATION

All participants will be asked if they would like to receive a summary of the research findings and those who would like to receive a report will be sent a plain English Summary at the end of the study.

Results of the study will be prepared for publication in September 2018. Researchers aim to submit to the Journal of Advanced Journal and to present findings to the European Dialysis and Transplant Nurses Association in 2018.

## CONFLICT OF INTEREST

No conflict of interest has been declared by the author.

## RESEARCH GOVERNANCE

Sponsorship and insurance for this study will be provided by the Royal Devon and Exeter NHS Trust.

## STUDY TIMELINES

Site approval September 2017–October 2017.

Recruitment and data collection October 2017–April 2018.

Data analysis December 2017–July 2018.

Publication and Dissemination September 2018.

## APPENDIX A

### A.1

**Table:**

Potential Core Questions
How do you view the renal nurse's role in discussing sexual issues with patients?
Can you tell me about a situation where you had to discuss something of a sexual nature with patients?
What does sexuality mean to you?
What do you think influences how you feel about discussing issues of a sexual nature with patients?
Prompts:
You discussed… what influences how you feel about this?
You talked about… could you give an example to illustrate that?
You talked about… how do you feel about that?
You mentioned…Could you tell me more about that?
